# End-Point Position Estimation of a Soft Continuum Manipulator Using Embedded Linear Magnetic Encoders

**DOI:** 10.3390/s23031647

**Published:** 2023-02-02

**Authors:** Carlos F. R. Costa, João C. P. Reis

**Affiliations:** 1 Departamento de Engenharia Mecânica, Instituto Superior Técnico, Universidade de Lisboa, 1049-001 Lisboa, Portugal; 2 Instituto de Engenharia Mecânica—IDMEC, Instituto Superior Técnico, Universidade de Lisboa, 1049-001 Lisboa, Portugal

**Keywords:** continuum robot, soft material robotics, flexible robots, compliant joint/mechanism, underactuated robots, tendon/wire mechanism

## Abstract

Soft continuum robots are compliant mechanisms that rely on a deformable structure in order to achieve a desired posture. One of the challenges in designing and controlling this type of robot is to obtain the necessary proprioceptive information without resorting to external sensors, like cameras or 3D positioning devices. This requires a reliable and repeatable sensor that can be embedded in the highly deformable structure, distributed along its length, without imposing a significant change to the overall stiffness. This paper presents design considerations and practical results of estimating the tip position of a soft continuum manipulator module using embedded linear magnetic encoders. Three flexible scales with incremental tracks and a magnetic pole pitch of 2 mm are embedded in the robot structure as passive tendons, and six pairs of Hall effect linear sensors are used to measure the relative displacement between points along the outer surface of the structure. The curvature and tip position are then estimated from these measurements. Results are compared with the ground truth measurement of the tip position provided by a commercial optical tracker system. Average error estimates lower than 2.0 mm, with 8.7 mm peak value, were obtained for a robot module with a motion span of approximately 100 mm.

## 1. Introduction

Robotic manipulators have found widespread utilization in industry and logistics, and are nowadays expanding their field of application to other areas like medicine, agriculture and services. At least in part, this expansion is a consequence of the development of sensors and controller devices that allow the robot to be more aware of its environment, and to assume tasks in collaboration with human operators while guaranteeing their safety. However, the mechanical structure of commercially available robots had little evolution during the past few decades. The rigid link serial chain structure, in its several kinematic variants, has remained the standard for high working volume and high dexterity applications. This kind of structure allows for high positioning precision and accuracy with relatively low control complexity, but it has the important limitation of requiring large workspace clearances in order for the robot to assume the postures required to execute a task.

**Figure 1:**
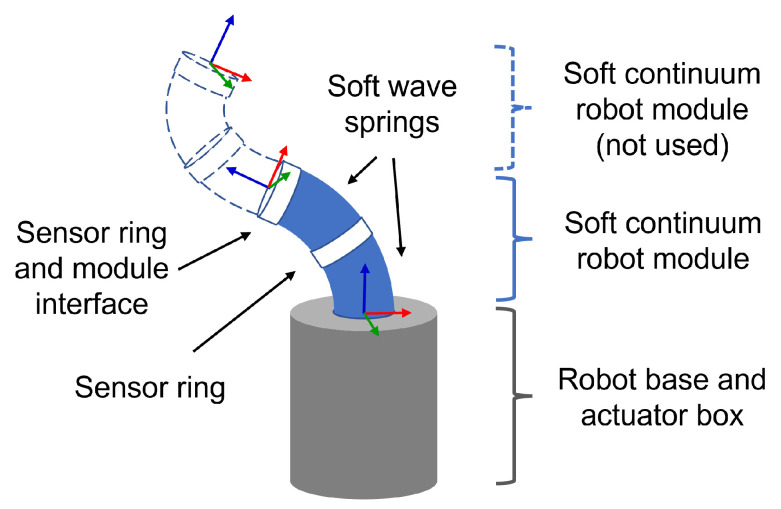
Depiction of the soft continuum robot. Arrows in green, red and blue represent base and tip axis x, y and z of each module respectively.

Continuum manipulator robots offer an alternative to the classical manipulator structure. They are composed of a set of deformable modules that are able to bend along the longitudinal axis, and in some cases also to extend and contract. For enhanced working volume and dexterity, the modules are usually assembled in a serial chain configuration like the one depicted in Figure 1. They are classified as under-actuated robots, because there will be a finite set of actuators controlling the infinite number of degrees of freedom (DoF) provided by the deformable structure. This also means that the robot will exhibit some form of passive mechanical compliance due to the non-actuated DoFs. Extensive research on these robots has been done in the past few decades and relevant literature reviews can be found for instance in [1,2,3,4]. Soft continuum manipulators (SCMs) have appeared in the literature more recently [5,6,7,8], and have the distinctive feature of including (sometimes exclusively) soft elastomer materials in the robot structure.

While one may argue that continuum manipulators are not ideal for precision or high-speed tasks like the ones frequently encountered in industry, the passive compliance and controlled deformability of the structure can be an advantage when working on collaborative tasks or in confined workspaces. The former can allow the robot to explore the environment tactilely, or interact safely with users without need for controller supervision (passive safety), while the latter allows the robot to adapt its postures to restricted or cluttered working environments. Both of these are relevant features, for instance, in assistive robots, that are meant to be deployed in a common household and interact with the user, and also do not require sub-millimetre precision to perform their tasks.

In order to fulfil the potential of continuum robots, the theoretically infinite number of DoFs of the structure prompts the need for more sophisticated proprioceptive sensing devices and control laws than the classic manipulator. Many examples in the literature resort to external cameras or 3D positioning devices in order to get precise information about the robot posture, for instance [5,6,8,9]. However, these sensors require an external mechanical fixture, and also in some cases an unobstructed view of the robot, thus limiting the advantages for work in confined spaces. This implies an obvious need for internal sensors that can provide proprioceptive information, with enough precision and reliability to estimate the robot posture, while maintaining the original advantages of the mechanical structure. In [10] a review of sensing technologies applied to soft robots is provided, where the authors classify sensors found in the literature according to the respective physical principles of operation. These are classified into five major categories, namely: resistive and piezoresistive sensors, capacitive sensors, optical sensors, magnetic sensors and also inductive sensors. The sensor implementation technologies can also be identified and include conductive liquids, conductive textiles, hydrogels, nanocomposite materials, optical fibres and piezoelectric film, amongst others. A more recent review focused on robots for surgical applications can be found in [11]. All these technologies and operation principles have their advantages and disadvantages. In general, sensors that depend on high deformations of a hyperelastic base material will tend to suffer from its drawbacks such as, for instance, significant hysteresis, highly nonlinear elasticity and long recovery times. This may also be the case for fluidic sensors that have channels embedded in such materials, or optical strain sensors. Temperature drift may also be a problem, for instance, for resistive sensors. Capacitive and magnetic sensors are especially sensitive to environmental variables like the proximity of conductive objects, externally induced magnetic fields and other sources of interference. Additionally, some of the physical operation principles are multidimensional in nature and decoupling of the desired measurement may be a difficult task. Such is the case, for instance, of capacitive and optical strain sensors that rely on the Poisson effect of a stretching material to measure longitudinal displacement, but are also sensitive to pressure applied in a perpendicular direction.

In more recent literature these and other methods have been used to estimate the shape of continuum robot structures. In [12] an inductance-based sensor is used to estimate the extension and contraction of the bellows that actuate each continuous module. A set of coils are wrapped around the bellows’ smaller diameter, and the inductance variation is measured. The modules are actuated in bending only, and have a neutral axis length of 19.7 cm. A root mean square (RMS) error of 1.11° regarding the orientation of a single module is reported, together with a 1.3 mm error for a lateral displacement of 14 mm when a perpendicular external load is applied at the distal end. Other concepts based on the variable inductance of coils or helical springs have been proposed in [13,14,15]. In [15], a helical spring sensor is used on a continuum robot with 140 mm length and 20 mm diameter actuated in bending only. A mean error of 5 mm in the desired radius of curvature is reported using open loop control, and is reduced to 2 mm when using closed loop control. In [16,17] magnetic sensors are used to detect the posture change of small permanent magnets embedded in the structure of the robots. In [16] the robot has two modules; the proximal module has a length of 100 mm and the distal module 95 mm, both with 30 mm diameter. The robot is actuated in bending only. Maximum and minimum errors of 9.339 mm and 0.227 mm, respectively, are reported for tip position estimation. In [17] the robot also has two modules, but the proximal module has a diameter of 53 mm and the distal module 41 mm. Both have the same length of 118 mm. The modules can be actuated in bending as well as extension/contraction. The robot is used to study the possibility of tactile exploration of the environment, and the sensor measurements are compared only with a theoretical model. Mean errors of ±2 mm are reported for this case when no obstacle is present. In [18] a resistive sensor composed of a conductive elastomer coiled around an elastic substrate is proposed. The sensor is implemented on a pneu-net actuator to estimate bending. In [19] a data-driven method for shape estimation of continuum manipulators using Fiber Bragg Grating optical sensors is proposed. The manipulator has a single module with 35 mm length and 6 mm diameter. The authors report a mean error of 0.11 mm estimating tip position for the data driven approach, that compares with a mean error of 1.9 mm for large deflections using a conventional deformation model. In [20] ionic liquid sensors are used to estimate the tip position of a continuum robot with two active segments, with bending capability only. Each segment has 13 mm diameter and 65 mm length. Estimation errors of 1.5% and 2.5% of the total length are reported for isolated motions and 3D trajectories, respectively. In [21] shape estimation of a continuum robot is obtained using passive tendons, a concept introduced in [22]. The robot structure has a single module with 115 mm length and a square section with 15 mm width, capable of deforming in bending and twisting. A total of 20 passive tendons are embedded in the structure, each tendon having one tip attached to the deformable section and the other tip free to displace longitudinally beyond the base structure of the robot. By attaching the passive tendons at different sections along the length of the robot, and different points in each section, their free displacement beyond the base provides information about the deformation of the module. When a curvature is imposed to the structure the tendon displacements are measured using cameras, and an estimation of the shape and tip displacement of the module is computed. In the three experiments reported, tip position errors of 3.6 mm, 2.5 mm and 4.1 mm were obtained by comparing with ground truth values directly measured by another camera. Twisting displacement was also studied, with a reported error of 6.4° for an imposed tip rotation of ≈45°.

With the exception of [21], recent literature seems to explore the same sensor principles and technologies mentioned in [10], albeit with different approaches to practical implementation and in some cases with improved results. In this work we adopt the concept of passive tendons, where the tendons are flexible magnetic scales similar to the ones used in industrial linear magnetic encoders. Encoder sensors are embedded in the robot structure following the path of each scale in order to measure the relative displacement between sensors. Knowledge of these distances allows the estimation of the curvature and tip position of one module. This approach requires only three passive tendons that can extend through any number of modules used in the robot, and does not require an additional apparatus at the base in order to measure the displacements. Because the magnetic scales slide almost freely inside the robot structure, drawbacks that usually would arise from high deformation of sensor base materials are not present. Interference from external magnetic fields can still affect the measurement. However, because the distance between sensors and scale is approximately constant, the amplitude and relative phase of the encoder signals is predictable and can be used, in principle, to minimize the influence of external noise. The main drawbacks of the proposed sensors are the ones associated with the utilization of passive tendons and permanent magnet materials in the robot structure. Namely, one needs to store the excess length of the passive tendons when the robot is in a contracted posture and the robot cannot operate in environments where the presence of ferromagnetic materials is not acceptable. These sensors were integrated in a SCM module based on the geometry of a wave spring with bending and extension/contraction capability. We present results of tip position estimation for active and passive deformation of the module and compare them against ground truth measurements obtained with a commercial 3D positioning system. The tip rotation along the central axis of the module cannot be directly estimated with this sensor arrangement and is not addressed in this work.

## 2. Materials and Methods

### 2.1. Manipulator Hardware Overview

The SCM robot used in this work has a single module with a tubular structure inspired by the geometry of a wave spring. Details on the development and fabrication of this design can be found in [23]. The current version of a single module is composed of two springs made from TPU material (Filaflex 82A, Recreus Industries, S.L., Elda, Spain) and three rigid rings made from PLA (PLA 4032D, Tucab Lda., Leiria, Portugal) as depicted in Figure 1. The module inner and outer diameters are 29.5 mm and 45.5 mm, respectively. Each spring section has a length at rest of 42 mm and the total length of the module is 120 mm. The rigid rings are used for tendon attachment, embedding sensors, and interfacing with the base or other modules. The robot structure is actuated both in bending and extension/contraction by a set of 3 tendons made from steel wire rope. The tendons are 1.1 mm in diameter and have one extremity attached to the distal end of the module while the other is displaced by the actuator. The tendons act as push/pull rods, meaning they can withstand load in compression as well as in tension. The actuator box is built from PLA material and houses 3 DC motors that drive the tendons through a mechanism of gearbox plus transmission belt. The belt is used to convert the rotary motion of the motor to a linear displacement, and drives the tendon inside a slotted tubular guide to avoid buckling of the tendon when pushed. The motor displacements are measured by multi-turn potentiometers, and are controlled in closed loop by PD controllers implemented on an Arduino Uno Rev3 board (Arduino S.r.l., Italia).

### 2.2. Deformation Modes

**Figure 2:**
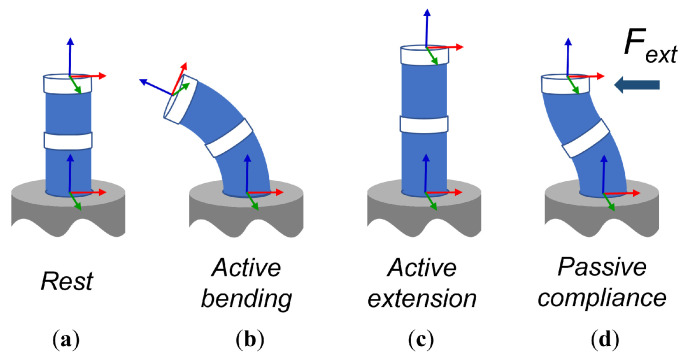
Relevant modes of deformation of the continuum robot module: (**a**) module depiction at rest for comparison, (**b**) module in controlled bending, (**c**) module in controlled extension/contraction, (**d**) module under influence of a radial force applied to the distal end. Arrows in green, red and blue represent base and tip axis x, y and z respectively.

Continuum manipulators are under-actuated robots that exhibit compliant elastic behaviour when some load drives the uncontrolled deformation modes. The significant deformation modes for a tendon-driven robot were thoroughly studied in [9] and examples are depicted in Figure 2. In our SCM module the tendons control bending and extension/contraction only. Two of the most relevant modes that are not controlled by our design are the torsional deformation and the radial displacement shown in Figure 2d). The wave spring geometry was chosen for its good torsional stiffness properties, that will limit the static positioning error when the robot is subjected to torsional loads. However, when a load is applied to the module along a radial direction, the continuum deformation kinematics of the structure will tend to respond with an ‘s’ shape posture. This is composed of two bending deformations in opposite directions in the upper and lower half sections of the module, respectively. In this case, one cannot resort to stiffening the structure design for improved positioning performance, because that would impose higher loads on the actuators to achieve bending, and an overall increase in cost, volume and energy consumption of the actuators just to displace the robot structure. However, one can produce an accurate measurement of the tip displacement and try to compensate the error using the controlled modes. For this, one needs an estimate of the radius of curvature for both the upper and lower sections of the module, that in our case can be obtained by measuring the relative displacement of the central ring along each tendon. This approach was already proposed by Felt et al. in [12] using inductance sensors and is implicit to the passive tendon approach used in [21]. Notice that this elastic displacement is not generated exclusively by external forces interacting with the robot. Any posture of the robot that deviates from the vertical direction will result in an elastic displacement generated by the radial component of the weight of the robot structure plus payload. In general, this effect will add some positioning error to the tip displacement directly induced by the controlled modes.

### 2.3. Kinematic Model

Constant curvature kinematics is a frequently used approach to the modelling of continuum robots [24]. This method assumes that the curvature of the neutral line of one flexible module is constant along the length, and also that section rotations due to torsion are negligible. Following the discussion in Section 2.2, in this work we will divide the module by the middle ring and consider two distinct radii of curvature, one for each flexible spring. In practice, this leads to the application of the constant curvature approach, as if each module was composed of two modules of smaller length, and adds to the number of model parameters in a way that can improve the accuracy of tip position estimation. In the following, the approach proposed in [25] will be used to derive the forward kinematics of the robot.

**Figure 3:**
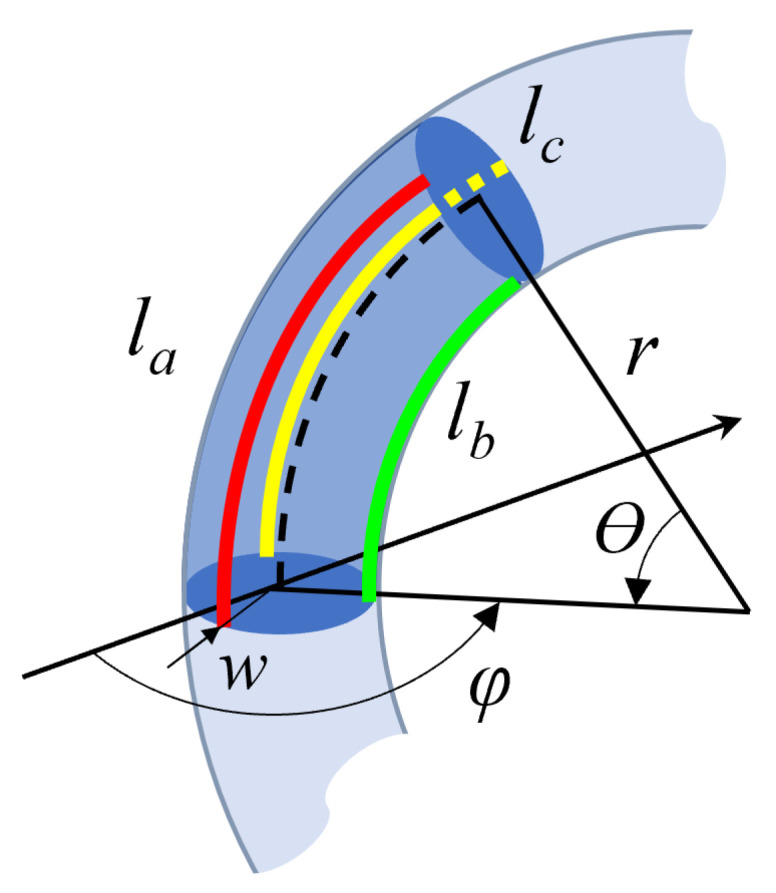
Parameters for a section of a constant curvature torus.

With reference to Figure 3, the radius of curvature *r*, angle of curvature θ and orientation of the bending plane ϕ, of a flexible section of the robot, can be computed from the arc lengths $l_{a}$, $l_{b}$ and $l_{c}$ of 3 tendons evenly spaced around the circumference of the cross-section through expressions (1)–(4) given by:

(1) $$\bar{l}=\frac{\left(l_{a}+l_{b}+l_{c}\right)}{3},$$

(2) $$\theta =\frac{2\;\sqrt{l_{a}^{2}+l_{b}^{2}+l_{c}^{2}-\left(l_{a}l_{b}+l_{a}l_{c}+l_{b}l_{c}\right)}}{3\;w},$$

(3) $$r=\frac{\bar{l}}{\theta },$$

(4) $$\phi =\arctan\left(\frac{\sqrt{3}\;\left(l_{c}-l_{b}\right))}{l_{b}+l_{c}-2\;l_{a}}\right),$$

where *w* is the constant distance of the tendons to the neutral line, measured in the plane of a cross-section of the torus.

Using these three parameters, the offset vector $p_{t}^{b}\;=\;{\left[x\;y\;z\right]}^{T}$ from the base reference frame to the origin of the tip reference frame can be computed from (5)–(7),

(5) $$x=\bar{l}\;\mathrm{sinX}\left(\frac{\theta }{2}\right)\;\cos\left(\frac{\pi -\theta }{2}\right)\sin\left(\phi -\frac{\pi }{2}\right),$$

(6) $$y=-\bar{l}\;\mathrm{sinX}\left(\frac{\theta }{2}\right)\;\cos\left(\frac{\pi -\theta }{2}\right)\cos\left(\phi -\frac{\pi }{2}\right),$$

(7) $$z=\bar{l}\;\mathrm{sinX}\left(\frac{\theta }{2}\right)\;\sin\left(\frac{\pi -\theta }{2}\right),$$

where **sinX** function is used according to [25] to avoid the singularity due to r→+∞ when the module is actuated in pure extension/contraction. This is given by (8)

(8) $$\mathrm{sinX}\left(\alpha \right)=\left\{\begin{array}{cc} 1 & \mathrm{if}\;\sin\left(\alpha \right)=0\;\wedge \;\left|\alpha \right|<\pi \\ \sin(\alpha )/\alpha & \mathrm{otherwise} \end{array}\right..$$

The rotation matrix can be obtained through the sequence of rotations in (9),

(9) $$R_{t}^{b}=R_{z}\left(\phi \right)\;R_{y}\left(-\theta \right)\;R_{z}\left(-\phi \right),$$

and finally the generic homogeneous transformation matrix $A_{t}^{b}\left(l_{a},l_{b},l_{c}\right)$ can be computed from (10):

(10) $$A_{t}^{b}\left(l_{a},l_{b},l_{c}\right)=\left[\begin{array}{cc} R_{t}^{b} & p_{t}^{b}\\ 0 & 1 \end{array}\right].$$

**Figure 4:**
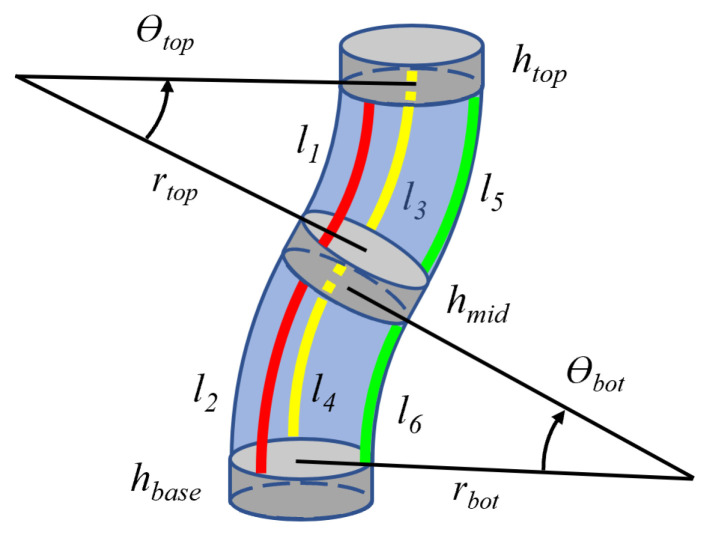
Constant curvature kinematics of the robot module.

With reference to Figure 4, the complete forward kinematics of the robot with one module can be expressed by a sequence of homogeneous transformations as given by Equation (11),

(11) $$T_{e}^{b}\left(l_{1},l_{2},\ldots ,l_{6}\right)=T_{0}^{b}A_{1}^{0}\left(l_{2},l_{4},l_{6}\right)T_{2}^{1}A_{3}^{2}\left(l_{1},l_{3},l_{5}\right)T_{e}^{3},$$

where $T_{e}^{b}\left(l_{1},l_{2},\ldots ,l_{6}\right)$ represents the transformation between the base of the robot and the tip of the module as a function of the arc lengths of each tendon $l_{1},l_{2},\ldots ,l_{6}$, $A_{1}^{0}\left(l_{2},l_{4},l_{6}\right)$ and $A_{2}^{3}\left(l_{1},l_{3},l_{5}\right)$ are the transformation matrices associated with the proximal and distal wave springs, respectively, (computed through the generic expression (10)), and $T_{0}^{b}$, $T_{2}^{1}$, $T_{e}^{3}$ are constant transformation matrices that express the offsets due to the thickness of the rigid rings. $T_{0}^{b}$ was added for convenience and is a function of the distance $h_{base}$ between the top base of the motor box and the bottom base of the proximal wave spring.

### 2.4. Linear Magnetic Encoders

Linear magnetic encoders are a type of displacement sensor that uses the spacial frequency of the permanent magnetic field of a base material—usually called a magnetic scale—to measure the relative position of a sensor that is sensitive to the intensity of the said magnetic field. Like optical encoders, the scale can be absolute or incremental, and can include additional tracks to provide index signals. Magnetic encoders can be designed to function in two distinct modes: a coarse measurement mode, where the magnetic poles are simply counted from a pulse train generated by on–off magnetic sensors; or a fine measurement mode, where the number of magnetic poles is complemented by analogue signals that provide a finer distance measurement within each pole pair. The latter are sometimes referred to as sinusoidal—or “sine–cosine”—encoders, and are commonly applied in industry in precision machinery.

**Figure 5:**
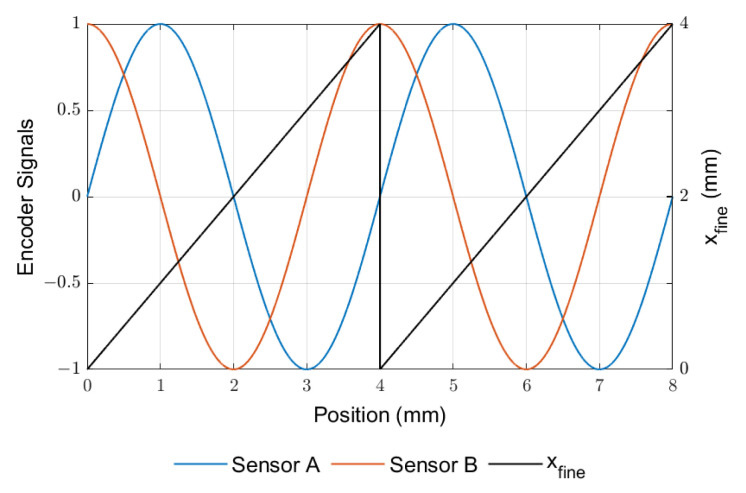
Ideal signals from sensor channels A and B of a sine–cosine encoder.

Quadrature signals are used by the encoders to determine the displacement direction. In practice, these consist of two distinct signals (referred to as channels A and B) that are spacially captured 90° out of phase with each other, hence the name “sine–cosine”. The ideal signal obtained from such an encoder is depicted in Figure 5.

The most relevant feature of a magnetic scale is called the *pole pitch*. This is the distance occupied by each magnetic pole along the scale length, that basically defines the resolution of the coarse measurement. The signals shown in Figure 5 refer to a pole pitch of 2 mm, that results in a spacial magnetic period of 4 mm encompassing a full north and south pole pair. From these signals, the position $x_{pos}$ at any time sample *k* can be obtained from (12) and (13):

(12) $$x_{pos}\left(k\right)=2P_{pitch}\times n_{period}\left(k\right)+x_{fine}\left(k\right)-x_{fine}\left(0\right),$$

(13) $$x_{fine}\left(k\right)=\left\{\begin{array}{cc} P_{pitch}\times \mathrm{atan}2\left(U_{sin}\left(k\right),U_{cos}\left(k\right)\right)/\pi & \mathrm{if}\;U_{sin}\left(k\right)\geq 0\\ P_{pitch}\times \left(2+\mathrm{atan}2\left(U_{sin}\left(k\right),U_{cos}\left(k\right)\right)/\pi \right) & \mathrm{if}\;U_{sin}\left(k\right)<0 \end{array}\right.,$$

where $P_{pitch}$ is the magnetic scale pole pitch, $n_{period}\left(k\right)$ is the current count of full magnetic periods travelled from a reference position and $U_{sin}\left(k\right)$, $U_{cos}\left(k\right)$ are the signals provided by sensor channels A and B, respectively. Function **atan2** represents the 2-argument, 4-quadrant arctangent that returns an angle between −π and π.

### 2.5. Encoder Manufacturing and Evaluation

Commercially available industrial grade magnetic scales are usually made from a magnetized rubber-like material, bonded to a metallic strip to ensure dimensional stability. Magnetic pole pitches ranging from 1 mm to 10 mm can easily be found from suppliers, but other values are also possible. However, the dimensions of commercially available scales make them difficult to integrate in our SCM robot, so we decided to develop our own scales from commonly available magnetized rubber sheets. This allowed us to tailor the dimensions, select a more appropriate reinforcement material and produce a double-faced magnetic scale with an approximately square cross-section, that has a much more homogeneous mechanical behaviour in multi-directional bending.

**Figure 6:**
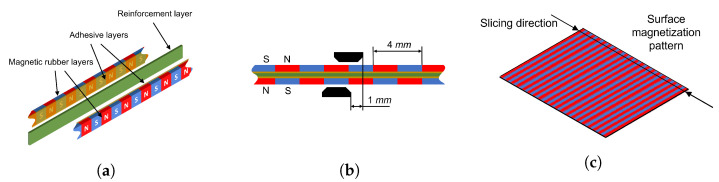
Depiction of the magnetic scale and relative sensor alignments: (**a**) scale composed of two magnetic rubber layers, with pre-applied adhesive and a reinforcement layer for added longitudinal stiffness; (**b**) top view showing the sensor offset distance and the spacial period of the magnetic poles (only the outermost magnetic layer is depicted for clarity); (**c**) magnetic rubber sheet pattern and slicing direction. The blue and red colours represent the magnetic South and North poles respectively.

Figure 6 shows a depiction of the magnetic scales developed for the robot. Each scale is composed of two magnetic rubber layers united back-to-back to a reinforcement layer (Figure 6a). The magnetic layers were cut from an A4 sheet with 0.8 mm thickness, in 2 mm width slices 250 mm in length. The reinforcement strip is a hard plastic material with a thickness of 0.2 mm, and so the cross-section of the scale is approximately square at 2 mm × 1.8 mm. Since the pole pitch is 2 mm, channel A and channel B sensors must have an offset of 1 mm between them to provide the sine and cosine signals. Two SS49E linear Hall effect sensors were used on each side of the scale to acquire the quadrature signals (Figure 6b). The magnetic rubber sheets from which the scales were sliced are polarized through thickness and at the surface the poles alternate in long stripes parallel to the shorter side of the sheet (Figure 6c). This is not the case for all commercially available sheets and care should be taken to identify the magnetization pattern before trying to replicate this manufacturing procedure.

**Figure 7:**
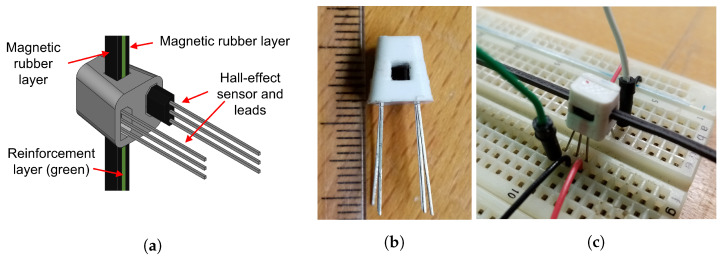
Encoder housing and single sensor setup: (**a**) 3D rendering with SS49E sensors; (**b**) housing implementation in PLA material (scale in mm); (**c**) experimental set up for single sensor tests.

A sensor housing was designed and 3D printed in PLA material to keep the appropriate relative distances between the sensor components. The housing has the approximate shape of a cube with 8 mm side, with a slight taper to fit into the distal ring of the robot. The 3D printing process has an announced precision of 0.1 mm, but this may be difficult to ensure due to the relatively small size of the parts. The orientation of the part during printing was selected in order to minimize the real practical tolerance of the distance between sensors, therefore minimizing the phase error between signals. Figure 7 shows a depiction and pictures of the encoder housing used for experiments, with the two Hall sensors and a section of the magnetic strip.

**Figure 8:**
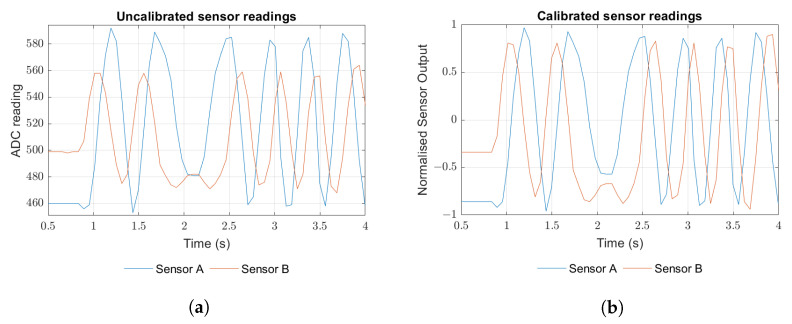

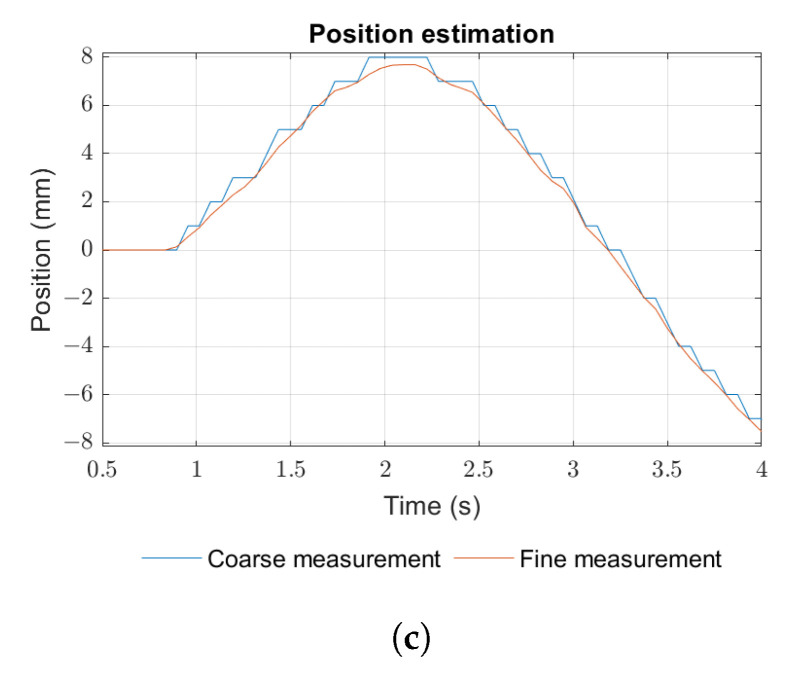
Encoder signals and reading: (**a**) raw channel signals, (**b**) normalized signals, (**c**) coarse and fine position readings from normalized signals.

An Arduino Mega 2560 (Arduino S.r.l., Italia) is used to read the analogue values from both channels. Figure 8a shows a typical raw ADC reading when the scale is moved relative to the sensors. The amplitude difference is constant and most likely caused by uneven distance from each sensor to the magnetic scale due to printing precision. For proper operation, each channel reading must first be normalized for the maximum and minimum amplitude of the sinusoidal signal that provides. Once appropriate offsets and scale factors are found (Figure 8b), the algorithm that implements Equation (12) provides $x_{pos}$ relative to a random initial position that is acquired as the first sample (Figure 8c).

**Figure 9:**
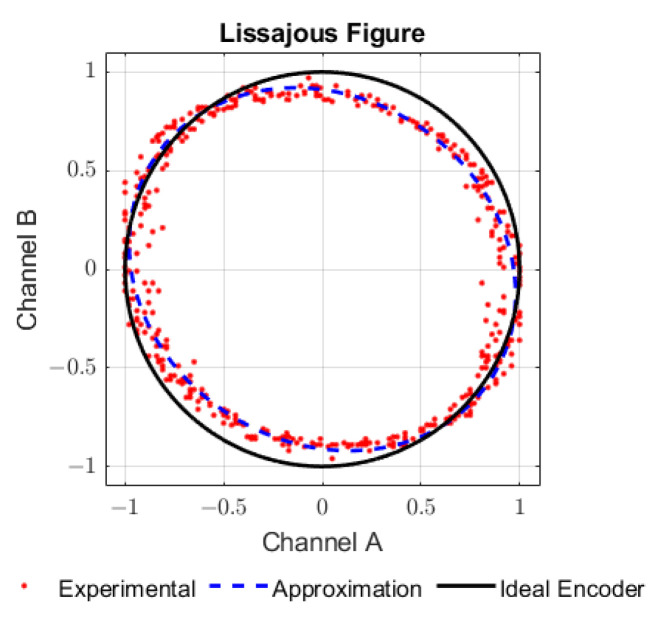
Lissajous figure of encoder signals.

In order to check the performance of the proposed encoder and manufacturing methods, the first step was to verify the phase difference between the two signals, that depends on the actual distance between sensors. Figure 9 shows a Lissajous figure plot of the sampled data against the circle that represents the 90° phase difference.

Experimental data are plotted as red dots and the blue dashed line is an approximation to fit the experimental data as given by Equation (14),

(14) $$\begin{array}{c} ChA=A\cos(\alpha +\varphi_{error})\;\\ ChB=B\sin(\alpha )\; \end{array}$$

where the best fit parameters were found to be *A* = 1, *B* = 0.90 and $\varphi_{error}$ = 4.72°. This angle represents 0.05 mm of actual positioning error between the sensors and is therefore within the expected manufacturing precision.

**Figure 10:**
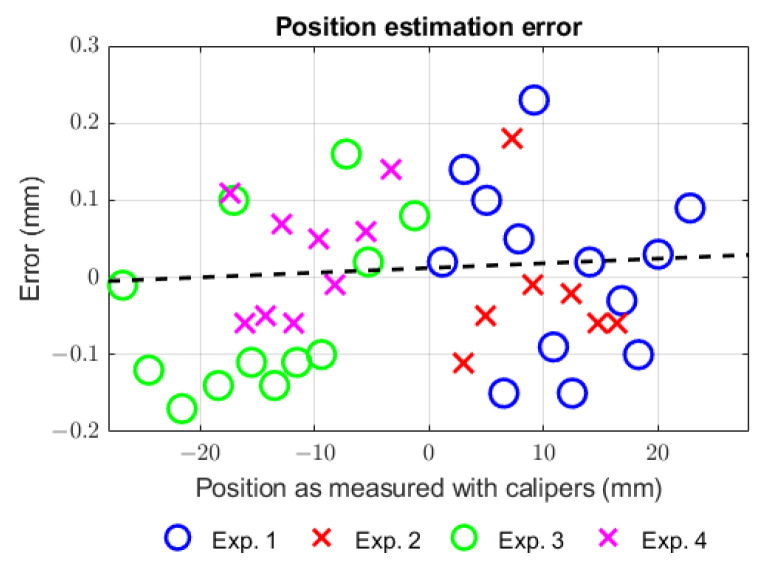
Position estimation error from measurements. The dotted line represents the linear regression.

To evaluate the accuracy of the encoder, a total of 40 random measurements were made across a range of −25 mm to +25 mm in the magnetic scale and compared with the measurement from a digital caliper accurate to 0.02 mm. For this procedure, the magnetic scale was attached to the caliper depth blade and measurements were manually registered from the digital display. Results are plotted in Figure 10, showing a mean position error of 0.01 mm with a maximum absolute error of 0.23 mm. A linear regression was computed for the data points showing a slope error of 1.2 mm/m. Commercial linear magnetic encoders have resolution and accuracies in the order of magnitude of μm, so these results do not compare well. However, the envisioned application of our encoder to a SCM module should produce tip positioning errors in the same order of magnitude of 1/10 mm, which seems good considering that SCMs are not intended for precision tasks.

### 2.6. Integration in the Manipulator Module

**Figure 11:**
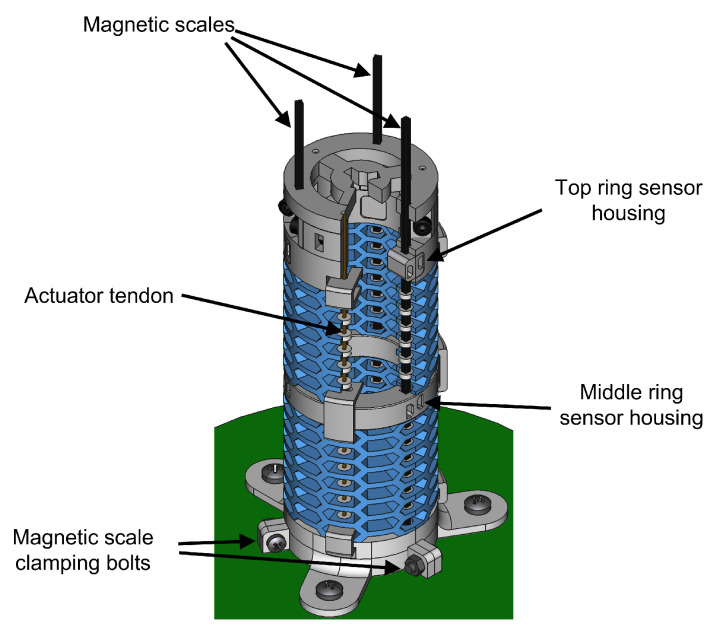
Cutaway view of the soft continuum robot module.

The complete proprioceptive system has 3 scales acting as passive tendons within the robot structure and a total of 6 encoders requiring 12 analogue readings. Figure 11 shows a cutaway view of the sensor implementation in the SCM robot module.

In this prototype we decided that the magnetic scales were to be attached to the base of the module instead of the distal ring, so that the excess length of the scales would come out at the top of the module. This allowed us to use an existing actuator box without the need for modifications to accommodate the excess length of the magnetic scales. The actuator push/pull tendons kept their attachment points as previously described.

**Figure 12:**
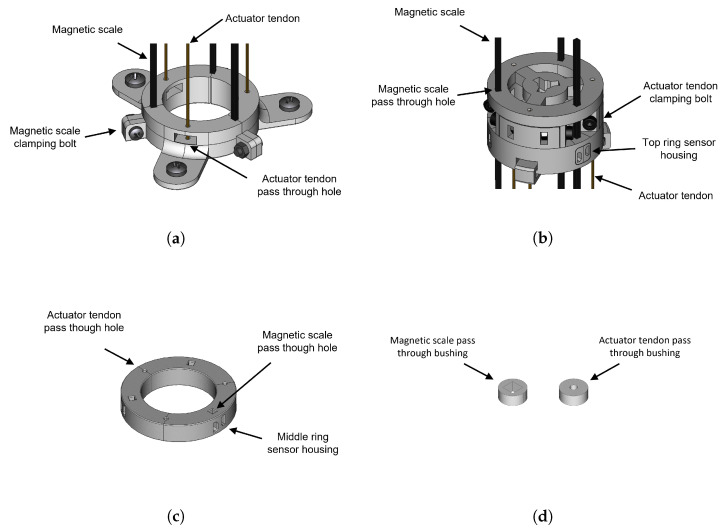
Rigid parts of the SCM robot module: (**a**) base ring, (**b**) distal ring, (**c**) middle ring, (**d**) pass-through bushing inserts (not to same scale).

Figure 12 shows details of the rigid parts of the robot that are 3D printed from PLA material. In Figure 12a the base ring is depicted, together with segments of the magnetic scales and the push/pull tendons. The ring is composed of three identical rigid parts at angles of 120${}^{\circ }$ that are attached together by bolts, the magnetic scales are clamped in between these parts while the push/pull tendons slide through the round holes with a nominal clearance diameter of 0.1 mm. Figure 12b shows the distal ring, where one can see one of the three encoder housings inserted into the outer face of the ring. The bolts are used to attach the push/pull tendons, while the magnetic scales slide through the square holes with a nominal clearance of 0.2 mm. Figure 12c shows the middle ring that is used to house the 3 encoder sensors for the mid section displacement measurements. Finally, Figure 12d shows the linear bushing inserts that are used in the wave springs pass-through holes to reduce friction from the sliding tendons. The attachment of the rigid rings to the soft springs is made through c-clamps also printed from PLA material, allowing the easy assembly and disassembly of the prototype.

**Figure 13:**
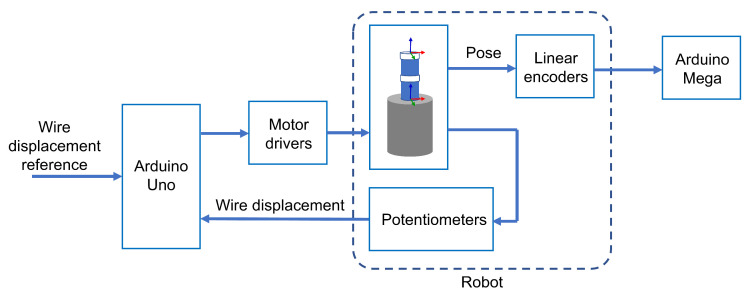
Schematics of the signal flow between hardware components.

**Figure 14:**
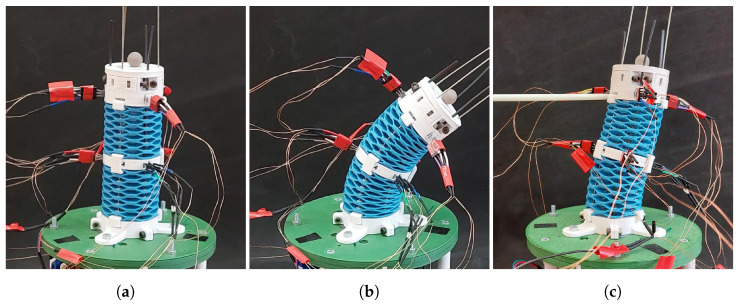
Robot prototype in selected postures: (**a**) rest (homing) position, (**b**) bent posture, (**c**) radial displacement resulting from an external force applied by the white rod to the left.

The Arduino Mega 2560 board was used to perform the readings and computations required for each encoder. The 12 analogue voltage values corresponding to each of the 6 encoders are read through ADC inputs and the displacements relative to a predefined homing position are computed onboard. The kinematic calculations to estimate the tip position were performed offline. Figure 13 shows a diagram of signal flow between hardware components of the robot and Figure 14 shows a picture of the prototype used in the experiments.

## 3. Results and Discussion

**Figure 15:**
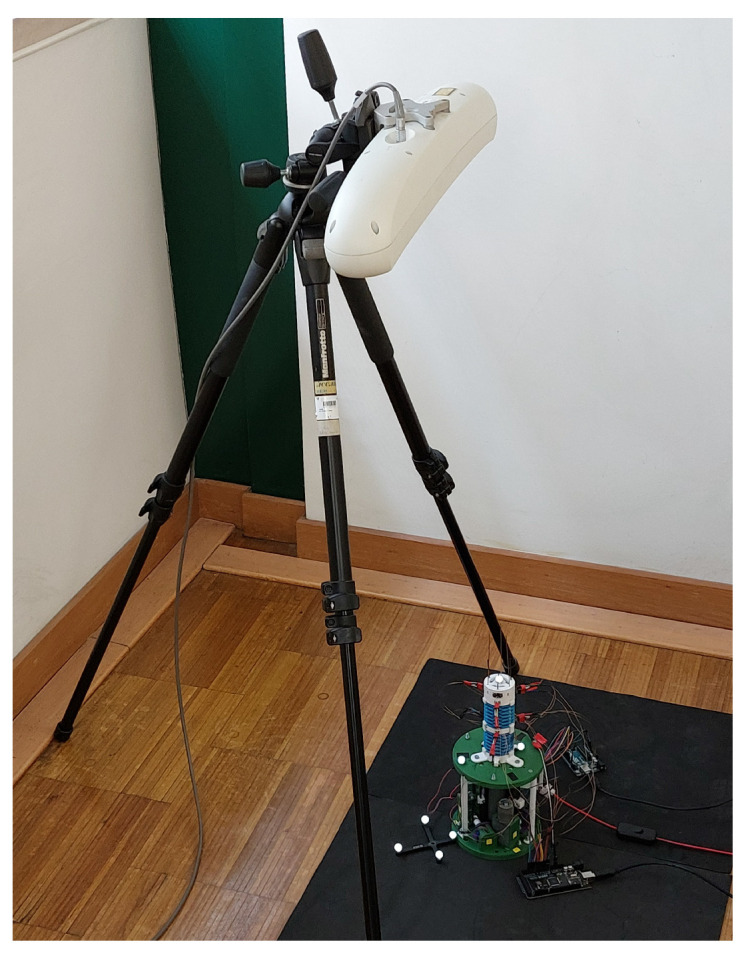
Experimental setup for data acquisition with the Polaris Spectra™ 3D Tracking System.

Two experiments were conceived in order to evaluate the performance of the proposed proprioceptive sensors. In the first experiment, the robot repeats a set of trajectories in open-loop using its own actuators. Open-loop in this case means that the tip position is not fed back to a tip position controller. However, the imposed length of each push/pull tendon is measured at the actuator box and the tendon displacement is controlled in closed-loop. A Polaris Spectra™ 3D Tracking System is used to detect the position of a single reflective marker placed at the tip of the robot and its results are used as ground truth for evaluation of the proprioceptive sensors. Figure 15 shows the experimental arrangement to ensure the line of sight between the Polaris sensor and the reflective marker at the tip. In the second experiment, sensor response to the passive compliance of the module was evaluated. Starting from the robot rest position, first a circular motion disturbance is manually imposed at the distal ring, then the robot is actuated in bending and again a disturbance motion is imposed around the new bent position. Sampling rate for both experiments was 24 Hz.

**Figure 16:**
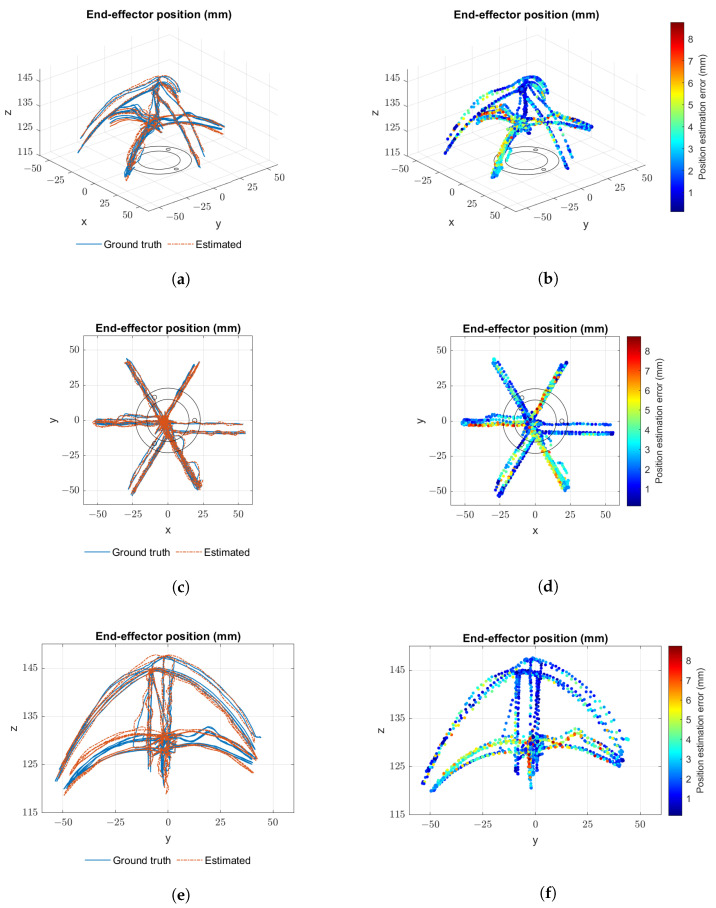
Different plot view points for open-loop trajectory results: (**a**,**c**,**e**) trajectory paths as perceived by the proprioceptive system (orange) and by the Polaris system (blue); (**b**,**d**,**f**) colour scale plot of the error between proprioceptive system readings and the Polaris.

**Figure 17:**
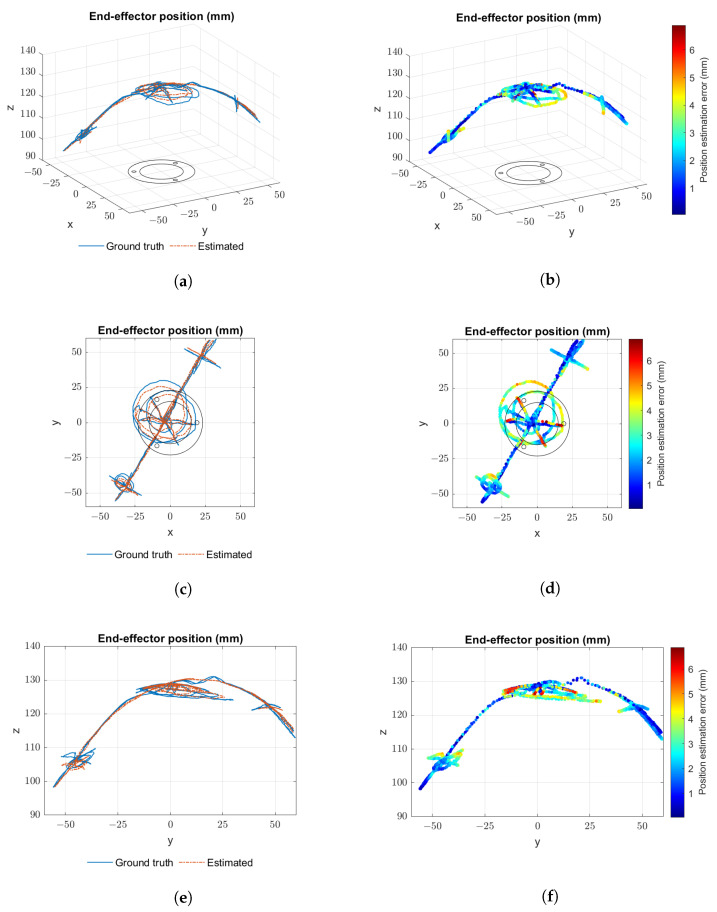
Different plot view points for passive compliance trajectory results: (**a**,**c**,**e**) trajectory paths as perceived by the proprioceptive system (orange) and by the Polaris system (blue); (**b**,**d**,**f**) colour scale plot of the error between proprioceptive system readings and the Polaris.

Figure 16 presents mixed data from several runs of the first experiment. Plots from Figure 16a,c,e show the paths taken by the end-effector from different viewpoints. Tip position as perceived by the proprioceptive sensor system is represented in orange and as perceived by the tracking system in blue. The paths explore the extended/contracted limits of actuation, and the lines that are closer to the z axis (with x≈0 and y≈0) represent simple open-loop extension and contraction movements. Plots from Figure 16b,d,f show a colour scale plot of the error between proprioceptive system readings and ground truth. The maximum absolute positioning error recorded was 8.7 mm. Figure 17 presents the same type of data for several runs of the second experiment, and the maximum absolute positioning error recorded in this case was 6.9 mm.

A summary of results taken from both sets of experiments is presented in Table 1. Results for the data points taken when the robot was stopped are presented separately and show a smaller error than those taken during motion. Mean positioning errors close to 2.0 mm were registered for both experiments.

## 4. Conclusions

The utilization of linear magnetic encoders for proprioceptive estimation of the tip position of a SCM robot was proposed and evaluated. A magnetic encoder prototype was developed, consisting of two Hall effect sensors and a custom flexible magnetic scale. For proper operation, a sensor calibration process was found to be necessary in order to normalize both encoder channel signals before processing. This calibration corrects for systematic errors due to mechanical manufacturing precision. Evaluation results show a mean position estimation error of 0.01 mm and a maximum absolute error of 0.23 mm. While these results do not compare well with commercial linear magnetic encoders, they are expected to produce tip positioning errors in the order of magnitude of 1/10 mm, which seems to be a good value for a soft manipulator module. If more accuracy is required it is possible to resort to magnetic encoder reading dedicated ICs. However, these may require specific pole pitches to be used and may not be so easy to integrate in the geometry of a SCM module because they are usually designed for single-sided scales.

A proprioceptive system consisting of six encoders using three magnetic scales was implemented in our SCM robot. An Arduino Mega 2560 board was used to read all analogue sensor signals and compute the relative displacement between the magnetic scales and the encoders in real time, showing promising results for future experiments with closed-loop control of the tip position. The overall accuracy of the proprioceptive system was investigated and compared against a ground truth measurement provided by a commercial 3D tracking system. Data from experiments where the robot follows a prescribed trajectory in open-loop revealed a mean position estimation error of 2.0 mm. A smaller error of 1.4 mm was obtained from the data points taken when the robot was stopped. The reason for this tendency is not clear from our experiments and requires further investigation. Data from passive compliance experiments shows a similar performance with a mean error of 1.9 mm and a maximum absolute error of 6.9 mm. The maximum absolute error recorded for the tip position during all experiments was 8.7 mm for a motion span of approximately 100 mm.

It should be made clear that the tip position estimation of a SCM module from the relative displacements of structural points depends not only on the sensor accuracy but also on the deformation model used for estimation. Mechanical clearances—used for instance in the pass-through bushings—or imperfections in the manufacturing of the wave springs can have an impact on the performance of the ideal constant curvature model. In this respect, improving the manufacturing precision, and using data-driven models instead of ideal geometric models, can probably contribute to improve even further the estimation accuracy. The results already achieved compare well with other solutions presented in the literature.

## Figures and Tables

**Table 1:** Summary of sensor evaluation results.

	Open-Loop Trajectory		Passive Compliance	
	Stopped	All Data	Stopped	All Data
Mean error (mm)	1.4	2.0	1.7	1.9
Median error (mm)	1.3	1.5	1.6	1.7
Max error (mm)	7.4	8.7	5.6	6.9
Mean abs. deviation (mm)	0.6	1.1	0.7	0.9
Data points	2435	3916	2057	3329

## Data Availability

Not applicable.

## Abbreviations

The following abbreviations are used in this manuscript:

DoF	Degrees of freedom
PLA	Polylactic acid
RMS	Root mean square
SCM	Soft continuum manipulator
TPU	Thermoplastic polyurethane
